# Differential effect of training impure tacts versus pure tacts plus intraverbal on the emergence of new verbal operants: A conceptual and methodological study

**DOI:** 10.3758/s13420-024-00636-1

**Published:** 2024-08-06

**Authors:** Miguel A. Maldonado, José Andrés Lorca-Marín, María Sheila Velo-Ramírez, Francisco J. Alós

**Affiliations:** 1 Departamento de Psicología, Facultad de Ciencias de La Educación y Psicología, Universidad de Córdoba, Calle San Alberto Magno, S/N, 14071 Córdoba, Spain; 2 Hospital Universitario “Reina Sofía”, Instituto Maimónides de Investigación Biomédica de Córdoba (IMIBIC), Cordoba, Spain; 3 Departamento de Psicología Clínica, Experimental y Social, Facultad de Ciencias de La Educación, Universidad de Huelva, Huelva, Spain; 4 Research Center in Natural Resources, Health and Environment” (RENSMA), Huelva, Spain; 5 Departamento de Psicología Experimental, Universidad de Sevilla, Seville, Spain

**Keywords:** Impure tacts, Intraverbals, Emergence, Training procedures, Compound stimuli, Verbal behavior, Order of presentation, Adults

## Abstract

The aim of this research was to test the effect of training impure tact versus pure tact and intraverbals on the emergence of new verbal operants (impure tacts), thus establishing a conceptual and methodological differentiation on these operants. This was done by varying the training order of intraverbal or impure tact to analyze and confirm whether or not impure tact is the mere sum of pure tact plus intraverbal and therefore has different functions and consequences in learning. An experiment was conducted with 30 participants randomly assigned to three groups. In Group 1, pure tact plus intraverbal and then impure tact were trained. In Group 3 the training order of these operants was counterbalanced. Group 2 was the control group, training only pure tact plus intraverbal. After the training phases, the emergence of impure tacts was tested. The results of this research indicate that the training of impure tacts favors the emergence of new impure tacts to a greater extent than the training of pure tact plus intraverbal and that they therefore have different functions. It is also shown that variation in the order of presentation of the type of training influences the subsequent emergence of new operants (impure tacts), so that creating a previous history of learning in impure tacts favors emergence even when the intraverbal alone is subsequently trained. This has important implications at both conceptual and methodological levels as it would contribute to the development of more effective language training technologies.

**Supplementary Information:**

The online version contains supplementary material available at 10.3758/s13420-024-00636-1.

## Introduction

The milestone that laid the foundations of what we know today as verbal behavior analysis was the publication of the book "Verbal Behavior" by Skinner in 1957. One of the most important approaches set forth in this book is that, to explain a given verbal behavior, it is necessary to analyze and determine the functional relationships that govern such behavior.

Following this assumption, other authors, such as Greer and Ross (2008), have continued the study of verbal behavior analysis and have laid the foundations for developing and training new verbal skills and operants. These authors define verbal behavior analysis as a subfield of basic and applied behavioral science, dedicated to identifying functional verbal repertoires and investigating training procedures to produce these when they are absent, further defining verbal behavior as behavior in which reinforcement is mediated by another person. Therefore, research should contribute to analyzing the function that different verbal operants may have and identify procedures to teach them (Fryling, 2017). This conception of language gives rise to understanding and implementing verbal behavior as an instructional technology that can be analyzed, measured, and controlled, and that can therefore be useful and effective for the training and production of language, which in turn may have a favorable effect on other more complex capabilities of human development.

In recent years, a considerable increase in the number of studies conducted and being published under this paradigm have been observed (Guerrero et al., 2020; Maldonado, 2020; Maldonado et al., 2020; Peña-Correal & Robayo-Castro, 2007; Pérez, 2016; Petursdottir, 2018). According to Schlinger (2008), this increase is due to the fact that behavior can be scientifically measured and the its interpretation is operational and parsimonious; also, the concepts proposed under this paradigm may be very useful and applicable for language training, especially for collectives with severe developmental limitations.

Despite all the studies published to date and the special relevance of verbal behavior for human development, there is currently some controversy regarding the definition of some of the verbal operants, their functions within language and the different procedures for training them (Alós et al., 2013; Belloso-Díaz & Pérez-González, 2015a, 2015b; Carp & Petursdottir, 2012; Greer & Ross, 2008; Guerrero et al., 2015, 2020; Maldonado, 2020; Maldonado et al., 2020; Pérez-González & García-Asenjo, 2016; Petursdottir, 2018; Petursdottir & Haflidadóttir, 2009; Petursdottir et al., 2008).

A starting point for the understanding of this field is the taxonomy presented by Skinner (1957), which included, among others, the operants known as *tact* and *intraverbal*. Tact is defined as a verbal behavior or operant that is evoked by the control of nonverbal stimuli, such as an object, a concrete event, or a property of the same, and is reinforced by generalized reinforcement (Greer & Ross, 2008; Michael, 1982; Skinner, 1957). The relevant operation of establishment is the deprivation of generalized reinforcement. Its acquisition may require positive reinforcement, but its emission may also be controlled by negative reinforcement. In addition, a highly relevant relationship is established with the discriminative stimulus, since this stimulus exerts the resulting control. A nuance within this operant is that it is also possible to work with an antecedent that is solely verbal, for example when tact is used to stimulate a verbal behavior. An example of tact is when a child says the word /pencil/ in the presence of the object and this response is socially reinforced. However, in the natural context of language, there are occasions when, for example, a person is shown a stimulus such as a pencil and subsequently asked what is it for, what is its shape, what is its color – to which the person responds according to the object and what has been asked and is then socially reinforced. Greer and Ross (2008) proposed a terminological differentiation regarding this operant: pure tacts, which occur under the control of nonverbal antecedents, and impure tacts, which occur under the control of verbal and nonverbal antecedents, such as asking /what is this?/ in the presence of another stimulus. This form of verbal operant could have great significance and importance in the expansion and development of language.

Impure tacts (verbal and nonverbal antecedent stimuli) and pure tacts (nonverbal antecedents), could be viewed as instances of multiple control proposed by Skinner (1957) to identify situations in which a single response can be elicited by several stimuli and a single stimulus can elicit several responses.

Axe (2008) and Michael et al. (2011) proposed the differentiation of convergent multiple control in which several stimuli are discriminative for the same response and divergent multiple control, in which a single stimulus can be discriminative for several responses. This framework could be of use in functionally differentiating pure tact from impure tact. In the case of pure tact, the antecedent stimulus may evoke several different responses. For example, when showing a child a pencil (yellow), the child may respond with /pencil/ (the name of the object) but could also respond with /paint/, /yellow/, /long/ (some property of the object) (multiple divergent control). In the case of impure tact, if we show the pencil and also ask about the color, there is more than one antecedent stimulus (in this case a verbal and a nonverbal stimulus) that can evoke only one correct response, in this case /yellow pencil/ (multiple convergent control).

The other operant of special relevance to this study is intraverbals. These are operants in which verbal responses are controlled by a vocal verbal antecedent, there is no point-by-point correspondence between stimuli and responses (Greer & Ross, 2008; Michael et al., 2011; Skinner, 1957). These can occur between people or for the same person. An example of this could be to ask for example /What is 2 multiplied by 2?/ and the participant's answer should be /4/.

Intraverbal skills are particularly relevant in terms of social relations. Therefore, they are a very important part of our complex language repertoire, and can be directly taught or can appear indirectly (emergence). In addition, these can be part of more complex verbal skills, involved, for example, in reasoning (Belloso-Díaz, 2016; Eikeseth & Smith, 2013; Pérez-González, 2019).

There are different types of intraverbals according to the number of elements or verbal antecedents involved and which control the response. Simple intraverbals include a relevant antecedent stimulus that controls the response, and complex intraverbals in which there is a combination of two antecedent stimuli that control a response (Belloso-Díaz, 2016). If we analyze this structure, the intraverbal response is in reality under the control of a compound stimulus (Alós et al., 2019; Guerrero et al., 2020). Other types of structures exist that are even more complex in this type of operant (see Eikeseth & Smith, 2013, for a review of the different types of complex intraverbal structures, and Aguirre et al., 2016, and Perez-Gonzalez, 2020, for a review of training of intraverbals).

In order to conceptualize and teach impure tacts and intraverbals it is important to determine whether these operants are part of the so-called compound stimulus.

In the case of simple stimuli, the following elements can be identified: A (e.g., an image or object), C (the possible response, e.g., the name of stimulus A), and finally the consequent stimulus. In this case, we would have simple discrimination (three-term contingency), since the stimulus that precedes the person's response is considered the antecedent or discriminative stimulus, and the effect it has on behavior is that it controls the probability of occurrence of certain responses. Finally, when the response preceded by the discriminative stimulus is emitted, the consequence or reinforcing stimulus appears. If we show a person an object, for example, a "violin," and he responds /violin/, his response is correct and the relationship will be reinforced. In this case the response /violin/ is under the control of the discriminative stimulus of the object "violin." The fact that the correct response is reinforced increases the probability of continuing to say the name each time the object is presented. According to the established relationship this would be a pure tact (non-verbal (discriminative) antecedent – response – consequence).

However, in compound stimuli the following elements can be identified: A (e.g., a picture or object), B (a word referring to a type of category such as "name" or property related to stimulus A), and C/D (e.g., the possible responses, e.g., the name of stimulus A or the name of the property related to A). In addition, the person is presented with two groups of stimuli (sets) related to two images or objects A1 and A2 from which the following relationships are deduced (A1B1 for which the response will be C1; A1B2 for which the response will be D1; A2B1 for which the response will be C2; and A2B2 for which the response will be D2). In these cases, the person to whom the compound stimulus is presented must perform a conditional discrimination, taking into account that stimuli A and B precede the response C/D, therefore, this type of discrimination is composed of two antecedent stimuli (discriminative and conditional), a response and a consequence. A given operant response in the presence of a discriminative stimulus will only be reinforced if it also attends to another antecedent stimulus, which, in this case, is the conditional stimulus. This last stimulus is the fourth term that would be added to simple discrimination to become conditional discrimination. If a person is shown two objects, for example, a "violin" (A1) and a "saxophone" (A2), and in addition, in the presence of one of these we add the word "name" (B1) or "family of instruments" (B2), the person must respond to the object in combination with the word presented, as the latter will condition the correct response. For example, if the picture "violin" is presented and "name" is added, the person must answer /violin/ (C1), but if the picture "violin" is presented and the word or phrase "instrument family" is added, the person must answer /string/ (D1). The latter are examples of impure tact. There is also the possibility of using a compound stimulus as a complex intraverbal, since if, for example, we present the word "family of instruments" (B2) and add the word "violin" (C1), the person must attend to the combination of these two stimuli (discriminative and conditional respectively) in order to correctly respond /string/ (D1). According to the established relationship, this would be a compound stimulus, which could be presented in the form of an impure tact (non-verbal antecedent (discriminative) – verbal antecedent (conditional) – response – consequence) or in the form of a complex intraverbal (non-verbal antecedent (discriminative) – non-verbal antecedent (conditional) – response – consequence).

Therefore, this type of stimulus differs from jointly presented stimuli and complex stimuli (see Alós et al., 2013; Guerrero et al., 2020); however, in the scientific literature there is no clear conceptualization of these operants under this classification of stimuli.

These types of structures with compound stimuli are more complex, allowing a greater number of stimulus combinations and, therefore, the possibility of further expanding the learning of new untrained stimulus relations, for example, facilitating greater emergence. This terminological clarification is also necessary to understand the functioning and implementation of impure tacts and complex intraverbals in training since these types of stimuli could be classified as compound stimuli.

Belloso-Díaz and Pérez-González (2015b) conducted a study to test the effect of tact or intraverbal training on the emergence of new intraverbals. They observed that both procedures are effective for the emergence of new intraverbal relations (although tact training was slightly more effective than intraverbal training). In this study there was no statistical support for the results, and at the conceptual level, no terminological difference was established between pure tact and impure tact. Some methodological aspects are the absence of counterbalancing of the sample and the training and testing of a reduced number of stimulus combinations, (see Belloso-Díaz, 2016; Eikeseth & Smith, 2013), nor tests of impure tacts, therefore, we understand that the functional differences of these operants and the implications this could have at the applied level are not fully elucidated. Guerrero et al. (2015) examined the influence of two training procedures using conditional and simple discriminations on the emergence of new tacts and intraverbals. In their study, the conditional discrimination and simple discrimination procedures (tacts) had similar effects in the training phases, but the simple discrimination (tacts) resulted in more derived relations (greater emergence).

However, this study has some methodological limitations, including the use of a small number of compound stimulus combinations and the use of simple intraverbals; the use of complex intraverbals could lead to a greater combination of stimuli and thus influence learning. Furthermore, no terminological differentiation was made regarding the use of pure or impure tacts, for example, they did not define the terms individually, using them interchangeably, when in fact they are not the same elements and do not have the same function. Recently, Maldonado et al. (2020) and Maldonado (2020) reported that training pure tact and intraverbal (Belloso-Díaz & Pérez-González, 2015a, 2015b) is not the same as training pure and impure tact (Alós et al., 2011, 2013; Guerrero et al., 2015), because training impure tact favors the emergence of new operants more than training intraverbals, it seems that both operants have different functions. In addition, a finding of particular relevance in these studies is that the creation of a learning history of impure tacts appears to improve performance on subsequent tests, even though training in later phases only includes pure tacts and intraverbals since impure tacts training produced superior performance (emergence) when testing impure tacts and complex intraverbals compared to intraverbal training (also see Guerrero et al., 2015; but see Belloso-Díaz and Pérez-González (2015b). However, in this study, the type of training was not counterbalanced among participants, therefore it is possible that there was a sequential effect that could have influenced participants' performance. This is a limitation at the methodological level that does not allow us to draw solid conclusions with high validity. To overcome it, different groups of stimuli (sets) should be presented in different presentation orders, for example, Set 1 followed by Set 2 with one type of training given to half of the participants and the opposite sequence to the remaining participants should be presented to adequately isolate the study variables. In this way, we could provide more robust data on the functional differentiation of operants and on whether the order of presentation can influence learning. Currently, there is controversy in the scientific literature as to which operant may facilitate greater and more effective learning of stimuli and relationships that have not been directly trained. Therefore, the analysis and study of these operants is a main focus of the present study. According to Greer and Ross (2008), of all the verbal operants, tact is the most important for language development, although these authors do not specify whether they refer to pure or impure tact. However, in the aforementioned studies by Maldonado et al. (2020) and Maldonado (2020), the authors explicitly state that impure tact can have important implications in the development and training of verbal behavior, favoring the expansion of verbal behavior through the emergence of new operants, which, in these studies, has only been proven to date in children.

The attainment of these language skills would enable the generalization, expansion, and facilitation of learning to many other relationships that are not directly taught. The process of emergence would be a very important developmental milestone that would enable the learning of new skills. This would have direct implications when training certain populations such as children with special needs (Alós & Lora, 2007; Alós et al., 2011; Carnett et al., 2020; DeSouza et al., 2019; May et al., 2013; Sautter & LeBlanc, 2006; Sundberg & Michael, 2001), favoring their development.

The present study aimed to contribute to the advancement of knowledge about the function, arrangement, and implementation of the different verbal operants involved in the expansion of verbal behavior, by means of effective training procedures, overcoming the limitations of the previous studies.

For this purpose, we tested the effect of training impure tact compared to pure tact and intraverbal on the emergence of new verbal operants (impure tacts), through an experimental design with a control group. Therefore, the aim of this research was to establish a conceptual and methodological differentiation between pure and impure tacts. This was done by assessing the emergence of novel verbal operants (new impure tacts) following exposure to training of pure tacts and impure tacts or of pure tacts and intraverbals, by changing the order of training across two sets of stimuli in a sample of adults. To date, no specific analysis has been conducted that studies the effect of varying the order of presentation of the type of training (impure tacts vs. intraverbals). This is of great importance, as it may allow for greater experimental control by varying the order of assignment of group conditions. The following hypotheses were derived from this objective:

- H1: The training applied by (1. Pure tact and intraverbal; and 2. Pure tact and impure tact) and by (1. Pure tact and impure tact; and 2. Pure tact and intraverbal) will produce a positive effect on learning, for example, a significantly higher number of correct trials in the post-tests of impure tact emergence compared to the pre-tests.
- H2: A significantly higher number of correct trials will be obtained in the post-tests of impure tact emergence after the training of pure tact plus impure tact than after the training of pure tact plus intraverbal, for example, the order of training of these operants will influence the acquisition of new operants.
- H3: The emergence of new impure tacts after intraverbal training will be higher due to previous training in impure tacts.

## Methods

### Participants

The sample comprised 30 participants (15 men and 15 women), who were students enrolled in the first year of the teaching degree at the University of Cordoba, aged between 20 and 27 years (*M* = 22.30 years; *SD* = 2.22 years). The participants were randomly assigned to one of the three experimental groups. All participants were equally distributed in each group by sex to avoid gender bias (five men and five women in each group), in Group 1 with a mean age of 22.52 years (*SD* = 2.01), Group 2 (control group) 20.92 years (*SD* = 2.00), and Group 3 23.21 years (*SD* = 2.16). The sample was probabilistic, randomly selected by means of a list of participants and randomly assigned to the experimental groups. Participants had similar socio-economic and educational levels and no diagnosed mental disorders.

### Materials and stimuli

Visual stimuli in the form of cards (6.9 cm × 9.2 cm) were used with four arbitrary images printed in black and white that were used as nonverbal stimuli (discriminative stimuli) and were administered manually by the experimenter.

In addition, verbal stimuli (conditional stimuli) were used in the form of three arbitrary words /alpha/, /beta/, /sigma/, pronounced vocally by the researcher. The stimuli used as participant responses were four arbitrary words, four alphanumeric words, and four numbers, /Cusa/, /Leca/, /Kote/, /Ralu/, /P20/, /Z10/, /F25/, /V15/, /111/, /222/, /333/, /444/. These verbal stimuli were also used as antecedent stimuli by the researcher in some of the experimental phases. The stimuli used in this experiment are shown in Table 1.

**Table 1.**
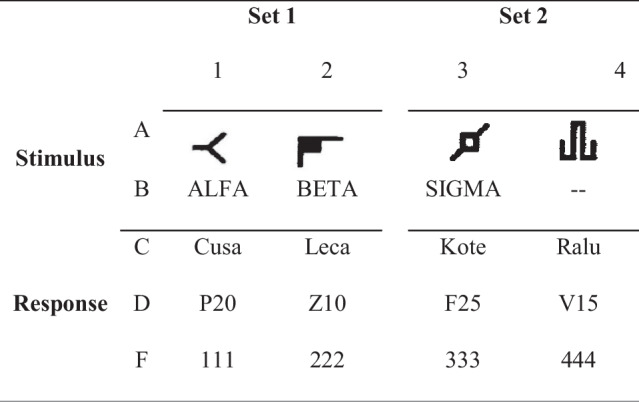
Stimuli used during the experiment

Each stimulus was designated by a capital letter and a number. The capital letters indicated the stimulus group (A, B, C, D, F), whereas the number (1, 2, 3, 4) indicated the order of the stimuli. In the alphanumeric code the number of the stimuli was only specified in cases where the discrimination included a single stimulus from a group, if it included any of the stimuli of the group it was indicated by "X." When the discrimination presented a verbal response, the letter "R" was added. For example, the arbitrary word /cusa/ as a stimulus was labeled “C1,” and when this was used as a response it was registered as “RC1.” Compound stimuli included two terms: AB.

Non-words (C, D, and F) could also appear as antecedent stimuli when intraverbals were trained, as follows; BX-CX-RDX, BX-DX-RCX, BX-CX-RFX, BX-FX-RCX.

In addition, a visual aid (a printout with the terms appearing horizontally) was used that included a row of all possible response stimuli for the participant, organized in an orderly fashion, for Stimulus Set 1 (Cusa, Leca, P20, Z10, 111, 222) or for Stimulus Set 2 (Kote, Ralu, F25, V15, 333, 444), depending on the phase of the experiment.

### Design

**Fig. 1:**
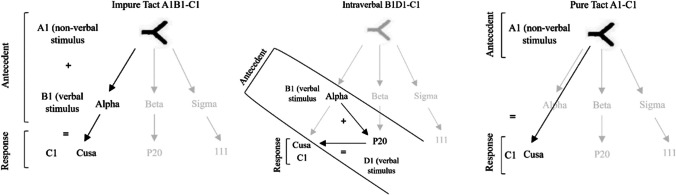
Example of trained relations in impure tact, intraverbal, and pure tact

A repeated-measures experimental design with a control group was carried out. In each of the three experimental groups, two sets of stimuli (Set 1 and Set 2) were used for the different training and testing phases (see Fig. 1). Groups differed based on the type of training performed: (a) Three pure tacts and four intraverbals in a first training phase, and three pure tacts and two impure tacts in a second training phase (Group 1); (b) three pure tacts and four intraverbals in a first training phase, and three pure tacts and four intraverbals in a second training phase (Group 2); (c) three pure tacts and two impure tacts in a first training phase and three pure tacts and four intraverbals in a second training phase (Group 3). Therefore, Groups 1 and 3 differed only in the order in which they received the training phases (the stimulus sets were maintained with Set 1 being used in the first training phase and Set 2 being used in the second training phase). Group 2 served as a control in which the same pure tact and intraverbal training was performed in both the first (Set 1) and second (Set 2) training phases. The effect of the intervention was measured in terms of the number of correctly emerged impure tact tests and the number of correct trials in the impure tact tests. Pre-test refers to the baseline carried out to test and rule out if there was prior learning from Set 1 and Set 2. Post-test 1 and post-test 2 phases refer to the tests carried out after training 1 and training 2, respectively. A summary of the experimental design is presented in Table 2.

**Table 2:** Common and specific phases (type of training) for Groups 1, 2, and 3

Group	Pre-tests		Training 1	Post-test 1	Training 2	Post-test 2
	Set 1	Set 2	Set 1	Set 1	Set 2	Set 2
1	IT	IT	Pure Tacts + Intraverbals	IT	Pure Tacts + Impure Tacts	IT
2	IT	IT	Pure Tacts + Intraverbals	IT	Pure Tacts + Intraverbals	IT
3	IT	IT	Pure Tacts + Impure Tacts	IT	Pure Tacts + Intraverbals	IT

Summary design. Antecedent events during training were pure tacts (e.g., A1-RC1), impure tacts (e.g., A1B1-RX), and/or intraverbals (e.g., B2C2-RD2). Pre- and post-tests did not include feedback; IT = impure tacts. The only difference between Group 1 and Group 2 was the change in the order of application of the verbal operants training of pure tact and intraverbals vs. pure tact and impure tact in Training 1 and Training 2. In Group 2 pure tact and Intraverbals were applied in Training 1 and Training 2

### Procedure

#### General procedure

The experiment took place in a quiet, isolated room, where the researcher and the participant were seated facing each other. First, the participant completed an informed consent form with personal data, specifying that participation in the study was voluntary and confidential and that the data collected during the study would be treated anonymously. The form also specified that the session would be audio-recorded for data collection and subsequent reliability of the data with another researcher. The following formula was used to calculate agreement: agreements divided by agreements plus disagreements multiplied by 100. The observers reached 100% agreement on all trials in all sessions with all participants. All experimental procedures were approved by the Human Research Ethics Committee of the University of Córdoba.

Participants were informed that they were going to participate in an experiment in which they had to give certain vocal verbal responses when picture cards were shown. Before the experiment began, each participant received the following instructions: *"Thank you for your participation in this experiment. In this experiment, sometimes I will show you one of these four figures* (showing the participant A1, A2, A3, and A4 as an example) *and say the name of a Greek letter /alpha/, /beta/, or /sigma/. Other times these Greek letters will be accompanied by the words and numbers /Cusa/, /Leca/, /P20/, /Z10/, /111/, /222/ or /Kote/, /Ralu/, /F25/, /V15/, /333/, /444/, and you will have to say one of these same words or numbers* (pointing out to the participant the answers on the corresponding printed aid while being named as an example) *that you think is related to the figure and/or letter I am saying. Try to do the best you can. There will be times when I won't be able to tell you if you are doing it right or wrong and other times in the experiment when I will tell you if you are doing it right, but you should do your best."*

### Training phases

At the beginning of each training phase, the researcher provided the participant with a few pre-exposure trials (the number of pre-exposure trials varied according to the specific training phase) that consisted of presenting the antecedent stimulus, the correct response as an example, and the participant's vocal verbal repetition of that response (which worked as a prompting strategy). During the training phases, the printed visual aid with the response stimuli of Set 1 or Set 2 (depending on the phase to which they corresponded) was present to the right of the participant, on the table at a distance of 50 cm. For example, if the pure tact operant was to be trained, the nonverbal stimulus (e.g., the A1 picture) would be presented followed by a vocal model (e.g., /cusa/), and then the participant repeated the answer (participant would echo the vocal model (e.g., echo /cusa/)). Training of the different operants consisted of presenting the antecedent stimuli allowing participants to produce a response, and delivering an outcome (reinforcement after correct responses or the application of correction for incorrect responses). In cases where the participant answered correctly, social reinforcement ("well done," "that's it," "very good," etc.) was provided vocally by the researcher, and the participant continued with the following trial.

When the participant made a mistake, a correction was applied by presenting the same trial once again, asking the participant to try again with a different answer, after which, if correct, reinforcement was given; if not correct, the trial was repeated again. This means that in these cases, during the correction, the researcher did not say the correct answer, instead, the trial was presented again with the antecedent stimuli used to give the participant a new opportunity to respond; if the correct answer was given this time, it was reinforced.

For example, if the stimuli (A1 + B1) were presented, for example, an impure tact through the presentation of the nonverbal stimulus or A1 picture plus verbal stimulus or word /alpha/, the participant's response had to be (C1), in this case, a vocal verbal response through the word /cusa/. In the case of intraverbal, if the stimuli (B1 + D1) were presented, for example, verbal stimulus /alpha/ plus verbal stimulus /P20/, the participant had to respond through the vocal verbal stimulus (C1), in this case the word /cusa/. Finally, in the case of pure tact, if the nonverbal stimulus (A1) was presented, the participant had to respond with the vocal verbal stimulus (C1), for example, the word /cusa/. The examples described can be seen in Fig. 1 below.

To advance from Phase 1 to Phase 2 of training, or from Phase 2 of training to testing, participants had to produce a correct response in ten consecutive trials. The experiment was terminated if participants reached 100 trials in either phase.

#### Test phases

Prior to the testing phases, it was specified that they would not be told whether the answer was correct or incorrect, although they were encouraged to try their best. Therefore, in the testing phases, the researcher presented the antecedent stimuli (all testing phases were impure tacts so the nonverbal stimulus or picture (A) and the verbal stimulus or word (B) were presented), the participant responded, and no consequence was applied. During these phases, the printed visual aid was not present. In the testing phases of the impure tacts, in order to consider that the operant had emerged, the learning criterion was that the participant responded correctly to ten or more trials (not necessarily consecutively) of the 12 trials presented in each phase. Before the test phases were carried out, the printed aid was removed.

#### Group-specific phases

The only difference between Group 1 and Group 2 was the change in the order of application of the verbal operants training of pure tact and intraverbals versus pure tact and impure tact in training 1 and training 2. In Group 2 pure tact and Intraverbals were applied in training 1 and training 2.

A description of the phases of each condition of the experiment for the three groups can be found in the Online Supplementary Materials (OSM) through the following link on the Open Science Framework (OSF): 10.17605/OSF.IO/T5SZC

### Data analysis

The responses recorded during both the training and the test phases were entered into a database, using the SPSS version 25 software, in which the relationships between phases and stimuli were previously established. Information on age, sex, and the group to which the study participants was randomly assigned was also included. First, a descriptive analysis of the data on the number of correct trials performed in each group (emergence of impure tact trials), as well as the total number of correct trials in the impure tact trials described in percentages for each group, was performed. Subsequently, an inferential analysis was performed through a repeated-measures mixed ANOVA (after verifying the sphericity and homogeneity of variances assumptions) with groups (1, 2 and 3) as between-subject factors and Set (1 and 2) as within-subject factors in the impure tact tests (pre-test and post-test) to check the existence of differences in the means and possible interaction effects. From this analysis it was possible to extract a within-group analysis and a between-group analysis to find possible statistically significant differences between the impure tact tests of the three groups of the experiment.

## Results

The results obtained individually for each of the three groups performed in this experiment are shown in Tables A1, A2, and A3 of the Appendix in the OSM (10.17605/OSF.IO/T5SZC).

**Fig. 2:**
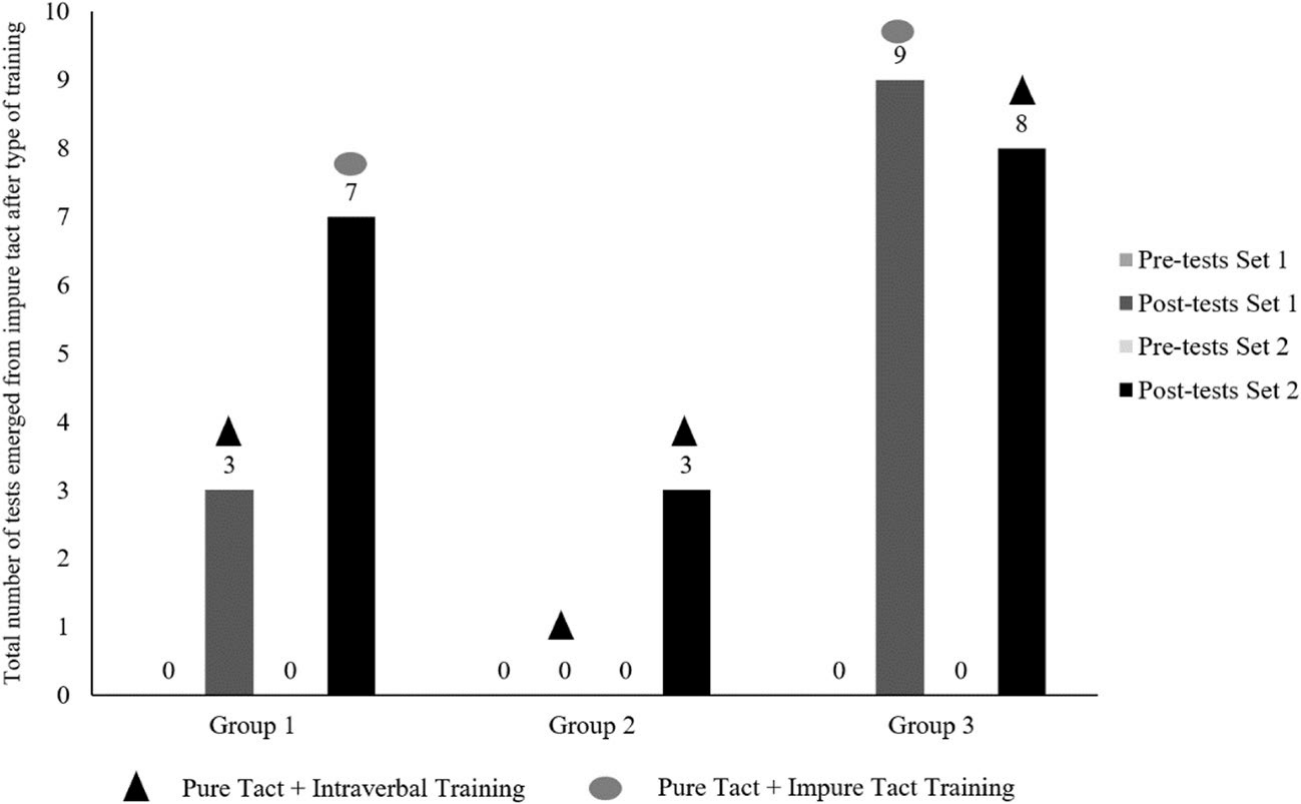
Number of tests emerged from impure tacts after type of learning (tests in which participants responded correctly to a number of trials ≥ 10 after the type of training applied) in the pre-tests and post-tests of Set 1 and Set 2 of all participants in each of the three groups

The data were analyzed to compare differences within and between procedures. As shown in Fig. 2, during the pre-test the three groups averaged 0/10 correct impure tacts for both Set 1 and Set 2. Therefore, prior to the training phases, no learning took place in the participants, which could be taken as a baseline.

Regarding the data obtained in the post-tests of the three groups, these can also be seen in Fig. 2. Group 3 has the highest emergence of impure tacts compared to Groups 1 and 2.

**Fig. 3:**
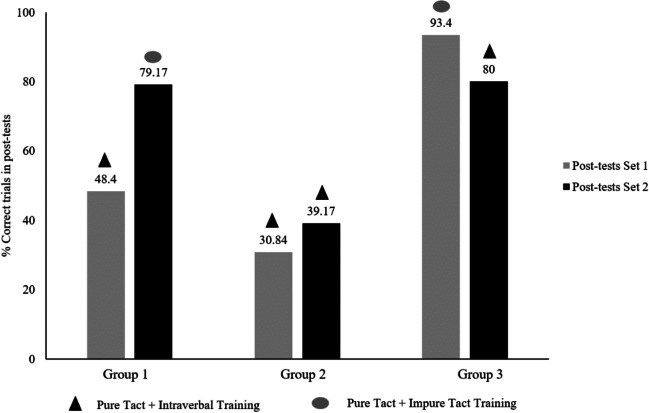
Percentage of emergence according to the number of correct trials in the impure tact post-tests of all participants in each group

Moreover, taking into account the number of correct trials in the impure tact tests out of the total number of participants in each group (120 trials for each impure tact test out of the total number of participants in each group), Fig. 3 shows the percentages associated with these total correct trials. The number of total correct trials in the post-test of Set 1 and Set 2 were 58 and 95, respectively, in Group 1, 37 and 47 in Group 2, and 112 and 96 in Group 3. When analyzing collectively the number of correct trials of the impure tact tests of all participants in the post-tests of Sets 1 and 2 for all groups, as well as their corresponding emergence percentages, Group 3 shows the highest percentages.

Table 3 shows the descriptive statistics for each of the impure tact tests performed in the three groups.

**Table 3:** Descriptive statistics of the three groups in the impure tact tests (pre-tests and post-tests)

	Group	*M*	*SD*	*n*
Pre_test_Set 1	Group 1	2.00	1.15	10
	Group 2	2.80	1.03	10
	Group 3	2.40	1.17	10
	Total	2.40	1.13	30
Pre_test_Set 2	Group 1	2.60	2.07	10
	Group 2	1.80	1.03	10
	Group 3	2.10	1.79	10
	Total	2.17	1.66	30
Post_test_Set 1	Group 1	5.80	3.68	10
	Group 2	3.70	2.75	10
	Group 3	11.20	2.53	10
	Total	6.90	4.34	30
Post_test_Set 2	Group 1	9.50	3.72	10
	Group 2	4.70	4.57	10
	Group 3	9.60	3.89	10
	Total	7.93	4.57	30

*M* = mean, *SD* = standard deviation, *n* = number of participants

Finally, a repeated-measures mixed ANOVA was performed in which the assumptions of Mauchly sphericity (p > 0.05) and equality of variances or Levene's test for within-subjects factors (Set) (p > 0.05) were confirmed. The factors included in the analysis were the groups (1, 2, and 3) as between-subjects factor and the sets (Set 1 and Set 2) as within-subjects factor, for the post-tests.

The analyses performed indicate that there is a significant interaction of the factors Set × Group in the variable impure tact tests *F* (2, 27) = 7.01, *p* = 0.004, ƞ^2^ = 0.34.

There was no main effect of the within-subjects factor (Set) for the impure tact tests *F* (1, 27) = 3.20, *p* = 0.085, ƞ^2^ = 0.11.

As for the between-subjects factor (Group), there was a significant main effect *F* (1, 27) = 158.83, *p* = 0.000, ƞ^2^ = 0.41, so there are statistically significant differences in the tests (post-tests) of impure tacts according to group.

Multiple comparisons between the group factor (Groups 1, 2, and 3) and the Set factor (Set 1 and Set 2) in the pre-test and post-test indicate that:

In Group 1, there were statistically significant differences between the pre-test of Set 1 and the post-test of Set 1 (p = 0.007) and between the pre-test of Set 2 and the post-test of Set 2 (p = 0.000), which indicates that there is emergence in impure tacts and this is due to the training applied in both cases. However, although there was emergence in both post-tests, there were statistically significant differences between the Set 1 post-test (M = 5.80; SD = 0.3.68) and the Set 2 post-test (M = 9.50; SD = 0.3.72) (p = 0.006), with greater emergence of impure tacts in the latter test, which means that pure tact plus impure tact training was more effective than pure tact plus intraverbal training.

In Group 2, there were no statistically significant differences between the pre-test of Set 1 and the post-test of Set 1, nor between the pre-test of Set 2 and the post-test of Set 2, which indicates that there is no emergence in impure tacts and therefore the training applied in both cases was not effective. Similarly, there were no statistically significant differences between the post-test of Set 1 and the post-test of Set 2, which is reasonable since the same type of training was applied in both cases (pure tact plus intraverbal) to this control group.

In Group 3, there were statistically significant differences between the pre-test of Set 1 and the post-test of Set 1 (p = 0.000) and between the pre-test of Set 2 and the post-test of Set 2 (p = 0.000), which indicates that there is emergence in impure tacts and this is due to the training applied in both cases. However, there were no statistically significant differences between the post-test of Set 1 (M = 11.20; SD = 2.53) and the post-test of Set 2 (M = 9.60; SD = 3.89). However, although the differences at the statistical level were not significant, it should be noted that the mean number of impure tacts that emerged following the training of pure tact plus impure tact (post-test Set 1) was slightly higher than the emergence of these operants following the training of pure tact plus intraverbal (post-test Set 2).

Similarly, the multiple comparisons between the Group and Set factors indicate statistically significant differences between the groups in some of the Set 1 and Set 2 post-tests. These differences occur in the post-tests of Set 1 between Group 1 and Group 3 and between Group 2 and Group 3. There are also significant differences for the post-tests of the Set 2 between Group 1 and Group 2 and between Group 2 and Group 3. These data indicate that in the post-test of Set 1, the group that obtained a higher emergence for impure tacts was Group 3 (where pure tacts and impure tacts were taught before this test). In the post-test of Set 2, the group that obtained a higher emergence was Group 3 (where pure tacts and impure tacts were taught before this test). Table 4 shows these data in detail.

**Table 4:** Multiple comparisons of impure tact emergence tests with respect to groups, between-group analysis

Set (tests)	Group		*p*
Post-test Set 1	Group 1	Group 2	.397
		Group 3	.001**
	Group 2	Group 3	.000***
Post-test Set 2	Group 1	Group 2	.042*
		Group 3	1.000
	Group 2	Group 3	.037*

^*^ p < .05, ** *p* < .01, *** *p* < .001

Finally, a multiple comparisons analysis was performed between the Set 1 and Set 2 post-tests between the three groups. This comparison of the post-tests of Set 1 of one group compared with Set 2 of another group was carried out to test how the order of training (application of training first or second, e.g., training pure tact plus intraverbal and then pure tact plus impure tact and vice versa) could affect the emergence of impure tact.

The data indicate that there were statistically significant differences (p = 0.038) between the post-test of Set 1 of Group 1 (test conducted after the training of pure tact plus intraverbal in the first place) and the post-test of Set 2 of Group 3 (test conducted after training pure tacts plus intraverbal in the second place, since the first training was of pure tacts and impure tacts), with a higher level of emergence in Group 3. However, between the post-test of Set 2 of Group 1 (test conducted after training pure tact and impure tact in the second place, as the first training applied was pure tact and intraverbal) and the post-test of Set 1 of Group 3 (test conducted after training pure tact and impure tact in the first place) there was no statistically significant difference (in both cases there was a similarly high level of emergence). These data indicate that training pure tact and impure tact is more effective than training pure tact and intraverbal, and that training pure tact and intraverbal is only effective if pure tact and impure tact have been taught previously. This last fact is also confirmed by comparing the post-test of Set 2 of Group 2 (test carried out after training pure tact plus intraverbal in first and second place as the control group) with the post-test of Set 1 of Group 1 (test carried out after training pure tact and intraverbal in first place) in which no statistically significant differences were found (in both cases there was no emergence or little emergence), and by comparing the post-test of Set 1 of Group 2 (test conducted after training pure tact and intraverbal) and the post-test of Set 2 of Group 3 (test conducted after the training of pure tact and intraverbal in the second place, but the first training was pure tact and impure tact) in which statistically significant differences were found (p = . 001) with a higher emergence in Group 3. In other words, in the post-test of Set 2, Groups 2 and 3 had previously trained pure tact and intraverbal; however, in Group 3 there was a first training of pure tact and impure tact and in Group 2 there was not. This indicates that if only pure tact and intraverbal are taught or taught first (instead of pure and impure tact in the first place), the training is not effective for the emergence of new impure tacts. These data can be seen in Table 5.

**Table 5:** Multiple comparisons between groups on post-test of Set 1 with respect to post-test of Set 2

Post-tests	Group	Set 2		
		Group 1	Group 2	Group 3
		*p*		
Set 1	Group 1	–	.561	.038*
	Group 2	.001**	–	.001**
	Group 3	.248	.001**	–

^*^ p < .05, ** *p* < .01, *** *p* < .001

## Discussion

The results obtained in the present study indicate that when the impure tact was trained in a first training phase (Group 3) or in a second training phase (Group 1), it had a positive effect on learning, resulting in more correct trials in the post-tests of impure tact compared to the pre-tests; therefore, this hypothesis is accepted (H1).

On the other hand, pure tact and impure tact training (Groups 1 and 3) favor a better performance (higher number of correct trials) in the post-tests of new impure tacts than pure tact and intraverbal training (Group 2). Furthermore, the interaction effect found implies that the emergence of impure tacts in the tests performed is not the same in Stimulus Set 1 and 2 as the order of presentation of the type of training varied across the three groups.

Therefore, the order of training of these operants influenced the acquisition of new operants, so that the second hypothesis (H2) proposed in the study can be accepted. That there are differences between the Group 1 Set 1 post-test and the Group 3 Set 2 post-test, but not between the Group 3 Set 1 post-test and the Group 1 Set 2 post-test indicates an effect produced by the change in the order of training presentation. That is, higher results were obtained in the impure tact test of Set 2 of Group 3 compared to the impure tact test of Set 1 of Group 1, the only difference being the order of presentation of the training (in the case of Group 3, impure tact was taught first and then intraverbal, while Group 1 was taught first intraverbal and then impure tact). Another relevant fact is that in Group 3 the difference in the occurrence of new impure tacts between the post-test of Set 1 and the post-test of Set 2 is not statistically significant since the results after training one or the other operant were high in both cases; however, from a qualitative point of view, better results were obtained after training the impure tact than the intraverbal. Therefore, training the impure tact first and then the interverbal seems to favor the emergence of new impure tacts; however, training the intraverbal and then the impure does not seem to have the same positive effect, as is the case in Group 1.

In fact, control Group 2 confirms these conclusions, since training only pure tact and intraverbal does not seem to be sufficient for the emergence of new impure tacts, since it is the group that exhibited the lowest performance. This is particularly relevant since, in Group 3, obtaining higher results in the emergence of new impure tacts after training impure tacts (first) as well as after subsequent intraverbal training indicates that creating a previous history of response learning on impure tacts benefits the emergence of new impure tacts, even if subsequently only intraverbal training is applied. That is, Group 3 performed better on the emergence of impure tacts after intraverbal training than Group 1, because Group 3 was first trained on impure tacts. This confirms the third hypothesis of the study (H3), since the occurrence of impure tacts was favored by previous training in impure tacts. Until now, in the specialized literature, terms such as impure tact (Alós et al., 2013; Guerrero et al., 2015) or pure tact and intraverbals (Belloso-Díaz & Perez-Gónzalez, 2015b) had been used interchangeably, and therefore it was not clear whether impure tact had the same function as pure tact and intraverbal. The results reported by Maldonado et al. (2020) in children and the present experiment, with adults, seem to confirm (now with greater procedural rigor) that an impure tact is not the same as both operants, as the participants' performance is affected by the training procedure used. Therefore, the data indicate that there is an influence of the type of training on the emergence of new impure tacts, with the training of impure tacts favoring their emergence over intraverbals. Moreover, the present experiment shows that the order of presentation of the operants during training has a differential effect, for example, it is confirmed that creating a learning history by means of impure tacts favors the subsequent emergence of learning, even training intraverbal on its own at a later stage.

In this work, when impure tacts versus intraverbals are taught, in the different groups, participants displayed a better performance in the evaluation of impure tacts, whether explicitly taught or emerged, something that confirms the data found in previous experiments. However, this research revealed a particularly relevant finding, which had not been observed to date. In Group 3, after creating a training history for impure tacts (Set 1), the training of pure tacts and intraverbals (Set 2) produced a statistically significant increase in the performance of the participants, compared to those who do not have such a history. Therefore, it appears that impure tact (Set 2) is now derived from the training of pure tact and intraverbals. This is of great relevance, both conceptually and for application in learning. From a conceptual point of view, this procedure has made it possible to study the effect of the learning history and has shown that creating a history of responding to compound stimuli is a necessary prior repertoire, and that only by having this repertoire will a person, if exposed to new stimuli presented individually, be able to respond to combinations of stimuli. From an applied point of view, in order to teach students with language limitations, one could state that it is appropriate for them to be explicitly exposed to impure tact tasks, since mere exposure to pure tacts and intraverbals (separately) cannot guarantee that they will respond successfully to tasks that involve attending to combinations of two stimuli.

As stated by Schlinger (2008), it is necessary to continue advancing this line of research, considering the important implications in terms of possible improvements in the procedures for training verbal behavior. The implementation of impure tacts significantly improves the learning conditions for a more effective retrieval, compared to procedures where this operant is not trained. Therefore, these procedures are suitable for implementation in populations of people with limitations or children with autism spectrum disorders, thus facilitating the acquisition and expansion of learning (see Alós et al., 2011; Carnett et al., 2020; DeSouza et al., 2019; Groskreutz et al., 2010; May et al., 2013; Pérez-González et al., 2007; Watkins et al., 1989). In this sense, the improvement of training through more effective procedures, in which verbal behavior is taken as a conceptual and methodological basis, could have a very positive impact on student learning (Sundberg & Michael, 2001), and therefore, this is a training technology that favors the expansion and generalization of language, and, consequently, favors more complex skills such as reasoning (Belloso-Díaz, 2016; Eikeseth & Smith, 2013; Pérez-González, 2019), comprehension (Hjetland et al., 2020), multilingualism (Rubio-Alcalá et al., 2019), and social skills (Müller et al., 2020), among others, which are necessary from a point of view of human development. All this would have direct implications on the good psychosocial adjustment of people in contexts such as family, school, or community, in addition to behavioral and emotional psychological aspects (Valera-Pozo et al., 2020).

In this work, conceptual and methodological limitations of studies such as those of Alós et al. (2013), Belloso-Díaz and Perez-Gónzalez (2015b), Guerrero et al. (2015), and Maldonado et al. (2020) have been overcome. For example, a control group was included in order to isolate the study variables. Thus, the implementation of a procedure using a control group in which pure tacts plus intraverbals are taught in both training sets has enabled us to compare the data with other procedures that did use impure tacts in different training sets. In addition, the sample has been larger in each experimental group, therefore, the results and subsequent statistical analyses have allowed us to find data with greater statistical robustness. Moreover, the use of two different sets of stimuli has also enabled us to overcome one of the most important limitations of previous experiments (see Maldonado, 2020), since in this manner, impure tacts were not explicitly taught, rather, on the contrary, they emerged throughout the evaluation of Set 2 of Group 3.

However, in the present study, a possible limitation at the methodological level is the application of the printed visual aid during training. This aid may have functioned as a stimulus during these phases, producing some effect on the acquisition of tact during the training. This could be improved by establishing one group in which this visual aid is applied and another group in which it is not applied, to see the possible associated effects with respect to the acquisition of new operants.

In conclusion, it has been experimentally verified that the type of training influences the emergence of new impure tacts; it has been confirmed that the effect of the order of presentation of the operants during training creates a history of learning through impure tacts, favoring the subsequent emergence of learning, even after intraverbal training; and the functional differentiation of these operants has been analyzed and confirmed.

## Conflicts of interest

The authors have no competing interests to declare that are relevant to the content of this article.

## Ethics approval

All procedures performed in studies involving human participants were in accordance with the ethical standards of the institutional and/or national research committee and with the 1964 Helsinki Declaration and its later amendments or comparable ethical standards. This article does not contain any studies with animals performed by any of the authors.

## Consent to participate

Informed consent was obtained from all individual participants included in the study. Written informed consent was obtained from the parents.

## Consent for publication

All authors consent to the publication of this study.

## Supplementary Information

Below is the link to the electronic supplementary material.

Supplementary file1 (DOCX 748 KB)

## Data Availability

The datasets generated and/or analyzed during the present study as well as part of the procedure carried out are available via the Open Science Framework at: 10.17605/OSF.IO/T5SZC.
