# Supplementary material for: Differential effect of training impure tacts versus pure tacts plus intraverbal on the emergence of new verbal operants: A conceptual and methodological study
Source: Learn Behav. 2024 Aug 6;52(4):339–51. doi: 10.3758/s13420-024-00636-1 (PMC11628571; doi:10.3758/s13420-024-00636-1)
Supplement: Supplementary file 1 — Supplementary file1 (DOCX 748 KB) [file 13420_2024_636_MOESM1_ESM.docx]

**Differential Effect of Training Impure Tacts vs. Pure Tacts plus Intraverbal on the Emergence of new Verbal Operants: A Conceptual and Methodological Study**

**Supplementary Materials**

Miguel A. Maldonado

Departamento de Psicología, Facultad de Ciencias de la Educación y Psicología, Universidad de Córdoba, Spain.

Instituto Maimónides de Investigación Biomédica de Córdoba (IMIBIC)

- <https://orcid.org/0000-0002-9126-2596>

E-mail: [z62mahem@uco.es](mailto:z62mahem@uco.es)

José Andrés Lorca-Marín

Departamento de Psicología Clínica, Experimental y Social, Facultad de Ciencias de la Educación, Universidad de Huelva, Spain.

Research Center in Natural Resources, Health and Environment” (RENSMA).

- <https://orcid.org/0000-0003-0331-020X>

E-mail: [andres.lorca@dpsi.uhu.es](mailto:andres.lorca@dpsi.uhu.es)

María Sheila Velo Ramírez

Department of Experimental Psychology, University of Seville, Spain.

- <https://orcid.org/0000-0001-6236-4748>

E-mail: [mvelo@us.es](mailto:mvelo@us.es)

Francisco J. Alós

Departamento de Psicología, Facultad de Ciencias de la Educación y Psicología, Universidad de Córdoba, Spain.

Instituto Maimónides de Investigación Biomédica de Córdoba (IMIBIC)

- <https://orcid.org/0000-0002-7667-4971>

E-mail: [ed1alcif@uco.es](mailto:ed1alcif@uco.es)

**Methods**

**Procedure**

The following is a description of the phases carried out for each of the three groups in the experiment.

***Group-specific phases***

**Group 1. Training of Pure Tact Plus Intraverbals (Set 1) and Pure Tact Plus Impure Tact (Set 2).**

This experimental group consists of 16 phases, each consisting of a block of randomized trials.

The Set 1 and 2 pre-tests (2 phases), Set 1 post-test (1 phase), and Set 2 post-test (1 phase) phases of testing were composed of blocks of 12 randomized trials of impure tacts in each phase. The training phases in training Set 1 (7 phases) and training Set 2 (5 phases) were composed of blocks of 10 or 12 random trials, depending on the phase, although the total number of trials performed by the participants in each phase could vary depending on their execution, since they had to reach the established learning criterion to move on to the next training phase. In these training phases, printed visual aid was provided and at the beginning of each block, 2 or 3 pre-exposure trials were provided (depending on the phase). Table 1 summarizes the procedure used in Group 1.

**Table 1.** Operants, phases and types of trials of Group 1, application of consequences, pre-exposure trials per block, number of total trials and learning criterion

| Group 1 | | | | | |
| --- | --- | --- | --- | --- | --- |
| Operant | Phase/ Types of trials | Consequences | Pre-exposure trials | Randomized Trials  Presented | Learning criterion |
| Pre- tests Set 1 and Set 2 | | | | | |
| Impure tact Set 1 | *1.(AB)-RX* | No | 0 | 12 | 10 |
| Impure tact Set 2 | *2. (AB)-RX* | No | 0 | 12 | 10 |
| Training 1- Set 1 | | | | | |
| Pure tact | 3. A-RC | Yes | 2 | 10 | 10 |
|  | 4. A-RD | Yes | 2 | 10 | 10 |
|  | 5. A-RF | Yes | 2 | 10 | 10 |
| Intraverbals | 6. (B2C)-RD | Yes | 2 | 10 | 10 |
|  | 7. (B1D)-RC | Yes | 2 | 10 | 10 |
|  | 8. (B3C)-RF | Yes | 2 | 10 | 10 |
|  | 9. (B1F)-RC | Yes | 2 | 10 | 10 |
| Post-tests Set 1 | | | | | |
| Impure tact | *10. (AB)-RX* | No | 0 | 12 | 10 |
| Training 2- Set 2 | | | | | |
| Pure tact | 11. A-RC | Yes | 2 | 10 | 10 |
|  | 12. A-RD | Yes | 2 | 10 | 10 |
|  | 13. A-RF | Yes | 2 | 10 | 10 |
| Impure tact | 14. A3B-RX3 | Yes | 3 | 12 | 12 |
|  | 15. A4B-RX4 | Yes | 3 | 12 | 12 |
| Post-tests Set 2 | | | | | |
| Impure tact | *16. (AB)-RX* | No | 0 | 12 | 10 |

_Note: The capital letters indicated the stimulus group (A, B, C, D, F), whereas the number (1, 2, 3, 4) indicated the order of the stimuli. In the alphanumeric code the number of the stimuli was only specified in cases where the discrimination included a single stimulus from a group, if it included any of the stimuli of the group it was indicated by "X". When the discrimination presented a verbal response, the letter "R" was added._

During the impure tact test phases, the experimenter presented the participant with the visual stimuli by means of the corresponding card (A1, A2, A3 or A4, depending on the phase) in combination with the name of a Greek letter (the experimenter told the participant the name of a Greek letter, /Alpha/, /Beta/ or /Sigma/). Once the combination of both stimuli was presented, the participant had to give the response he/she believed to be correct (vocal verbal response). No corrections or consequences were administered in these phases.


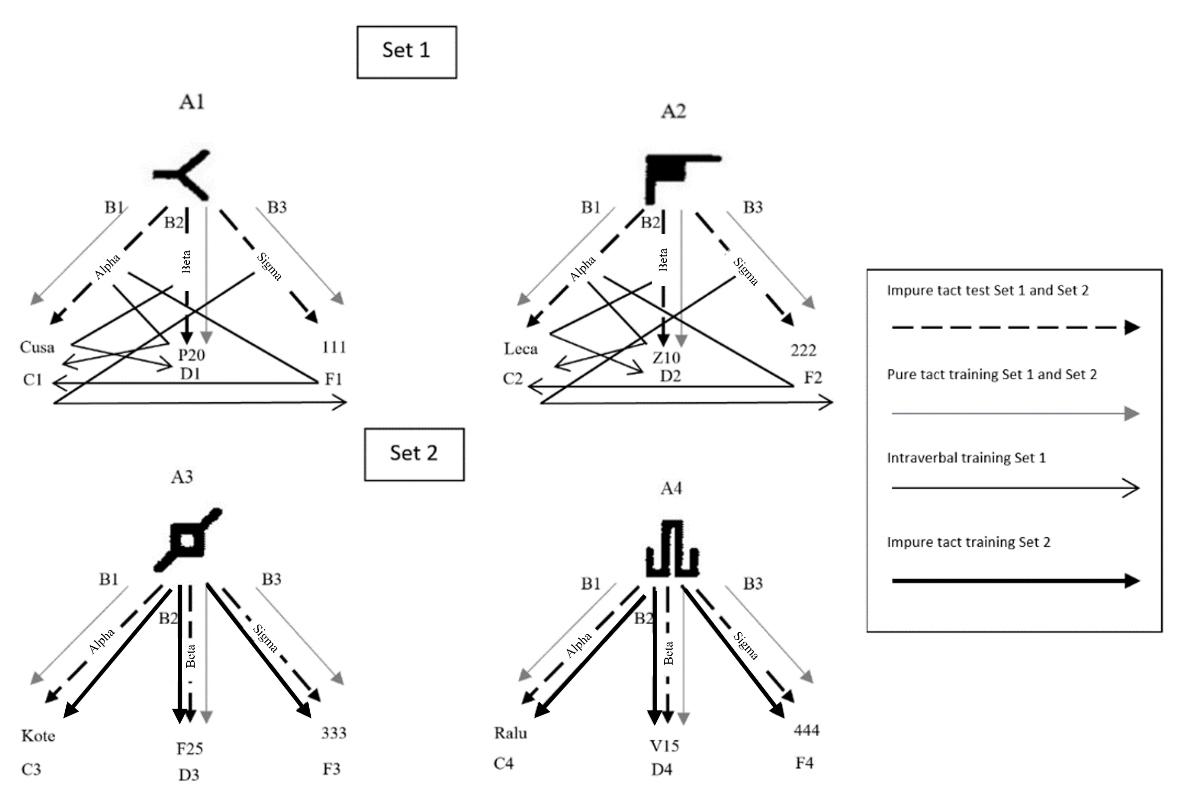
During the training phases, the printed visual aid was on the table, each phase began with the aid trials administered by the investigator, and the randomized training trials for each phase began to be presented. The researcher presented to the participant the antecedent stimuli (nonverbal stimulus, verbal stimulus, or a combination of both, depending on the phase) for the participant to subsequently give the response. Correct trials were reinforced and corrections were applied to incorrect trials. If the participant reached the learning criterion established in each phase, he or she moved on to the next phase. Figure 1 shows the trained relationships (solid lines) and tested relationships (dashed lines) during the different phases of the experimental Group 1.

**Figure 1.** Trained relations of pure tact and intraverbals from set 1 and pure and impure tact from set 2 (solid lines) and tested relations (dashed lines) during Group 1

**Group 2. Training Pure Tact Plus Intraverbals (Set 1) And Pure Tact Plus Intraverbals (Set 2).**

Group 2 constituted the control group for the experiment, comprised of 18 phases, each consisting of a block of randomized trials.

The pre-test Set 1 and 2 (2 phases), post-test Set 1 (1 phase) and post-test Set 2 (1 phase) phases of testing were identical to Group 1.

However, the difference between this procedure and the previous one is that the training phases of Set 1 (7 phases) and the training phases of Set 2 (7 phases) are repeated in the two training moments of this group, training the same operants in both sets of stimuli (3 pure tacts and 4 intraverbals). Therefore, this group acts as a control group. In addition, the training of Set 1 of Group 2 was identical to the training of Set 1 of Group 1. The trial structure, the criterion and the rest of the elements were identical to Group 1. Table 2 summarizes the procedure used in Group 2.

**Table 2.** Operants, phases and types of trials of Group 2, application of consequences, pre-exposure trials per block, number of total trials and learning criterion

| Group 2 | | | | | |
| --- | --- | --- | --- | --- | --- |
| Operant | Phase/ Types of trials | Consequences | Pre-exposure trials | Randomized trials presented | Learning criterion |
| Pre- tests Set 1 and Set 2 | | | | | |
| IDEM | | | | | |
| Training 1- Set 1 | | | | | |
| IDEM Group 1 (3 Pure tacts + 4 Intraverbals) | | | | | |
| Post-tests Set 1 | | | | | |
| IDEM | | | | | |
| Training 2 - Set 2 | | | | | |
| Pure tact | 11. A-RC | Yes | 2 | 10 | 10 |
|  | 12. A-RD | Yes | 2 | 10 | 10 |
|  | 13. A-RF | Yes | 2 | 10 | 10 |
| Intraverbals | 14. (B2C)-RD | Yes | 2 | 10 | 10 |
|  | 15. (B1D)-RC | Yes | 2 | 10 | 10 |
|  | 16. (B3C)-RF | Yes | 2 | 10 | 10 |
|  | 17. (B1F)-RC | Yes | 2 | 10 | 10 |
| Post-tests Set 2 | | | | | |
| IDEM | | | | | |

_Note: The capital letters indicated the stimulus group (A, B, C, D, F), whereas the number (1, 2, 3, 4) indicated the order of the stimuli. In the alphanumeric code the number of the stimuli was only specified in cases where the discrimination included a single stimulus from a group, if it included any of the stimuli of the group it was indicated by "X". When the discrimination presented a verbal response, the letter "R" was added; IDEM = it means that the pre-test and post-test of this group are the same as in Group 1; IDEM Group 1 (3 Pure tacts + 4 Intraverbals) = it means that this training phase (training 1) was the same as in Group 1._

*
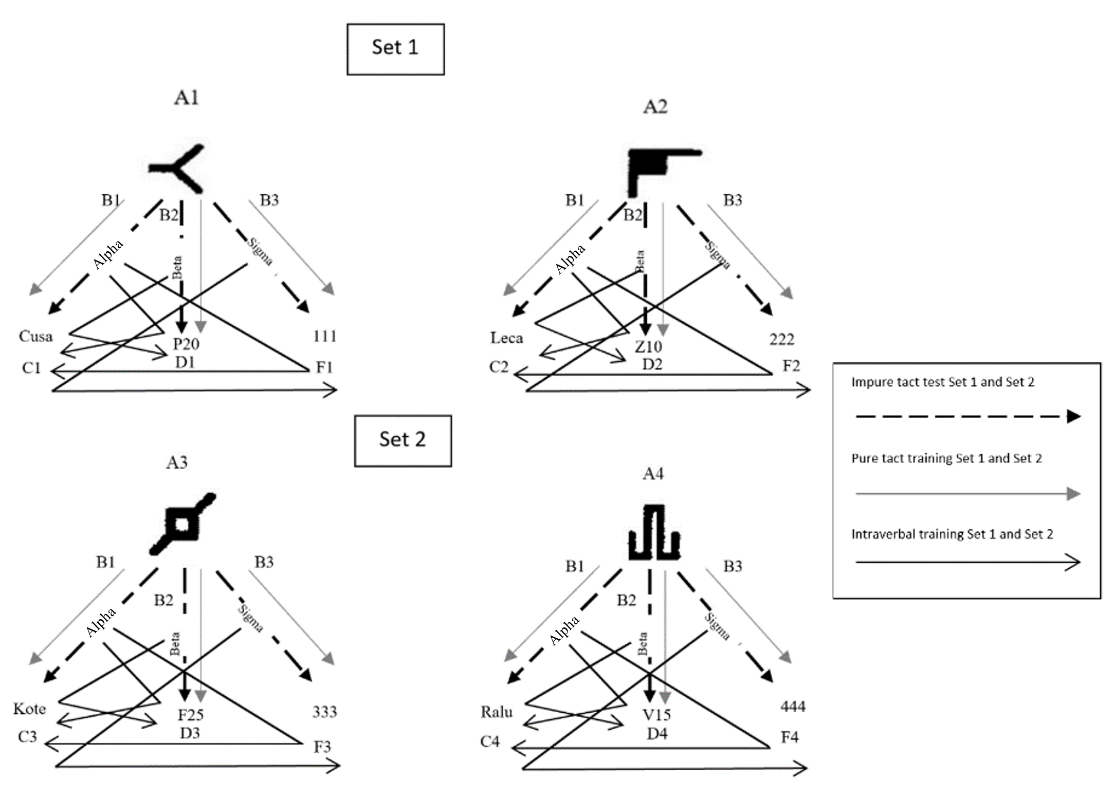
*Figure 2 shows the trained relationships (solid lines) and tested relationships (dashed lines) during the different phases of Group 2 of the experiment.

**Figure 2.** Trained relations between pure tact and intraverbals from Set 1 and pure tact and intraverbals from Set 2 (solid lines) and tested relations (dashed lines) during Group 2

**Group 3. Training pure tact plus impure tact (Set 1) and pure tact plus intraverbals (Set 2).**

**Phases.**

This experimental group consisted of 16 phases, each comprising a block of randomized trials.

The pre-test phases of Set 1 and 2 (2 phases), the post-test phases of Set 1 (1 phase) and the post-test phases of Set 2 (1 phase) were identical to those of Group 1 and 2.

However, the difference of this procedure in relation to the previous ones is that the training phases of Set 1 and Set 2 have been counterbalanced in Groups 1 and 3, i.e., they are identical, but the order of presentation of the type of training differs. The structure of the trials, the criterion and all other elements were identical to Group 1 and 2. Table 3 summarizes the procedure used in Group 3.

**Table 3.** Operants, phases and types of trials of Group 3, application of consequences, pre-exposure trials per block, number of total trials and and learning criterion

| Group 3 | | | | | |
| --- | --- | --- | --- | --- | --- |
| Operant | Phase/Types of trials | Consequences | Pre-exposure trials | Randomized trials presented | Learning criterion |
| Pre- tests Set 1 and Set 2 | | | | | |
| IDEM | | | | | |
| Training 1- Set 1 | | | | | |
| Pure tacts | 3. A-RC | Yes | 2 | 10 | 10 |
|  | 4. A-RD | Yes | 2 | 10 | 10 |
|  | 5. A-RF | Yes | 2 | 10 | 10 |
| Impure tacts | 6. A1B-RX1 | Yes | 3 | 12 | 12 |
|  | 7. A2B-RX2 | Yes | 3 | 12 | 12 |
| Post-tests Set 1 | | | | | |
| IDEM | | | | | |
| Training 2 - Set 2 | | | | | |
| IDEM Group 2 (3 Pure tacts + 4 Intraverbals) | | | | | |
| Post-tests Set 2 | | | | | |
| IDEM | | | | | |

_Note: The capital letters indicated the stimulus group (A, B, C, D, F), whereas the number (1, 2, 3, 4) indicated the order of the stimuli. In the alphanumeric code the number of the stimuli was only specified in cases where the discrimination included a single stimulus from a group, if it included any of the stimuli of the group it was indicated by "X". When the discrimination presented a verbal response, the letter "R" was added; IDEM = it means that the pre-test and post-test of this group are the same as in Group 1 and Group 2; IDEM Group 2 (3 Pure tacts + 4 Intraverbals) = it means that this training phase (training 2) was the same as in Group 2._

Figure 3 shows the trained relationships (solid lines) and tested relationships (dashed lines) during the different phases of experimental Group 3.


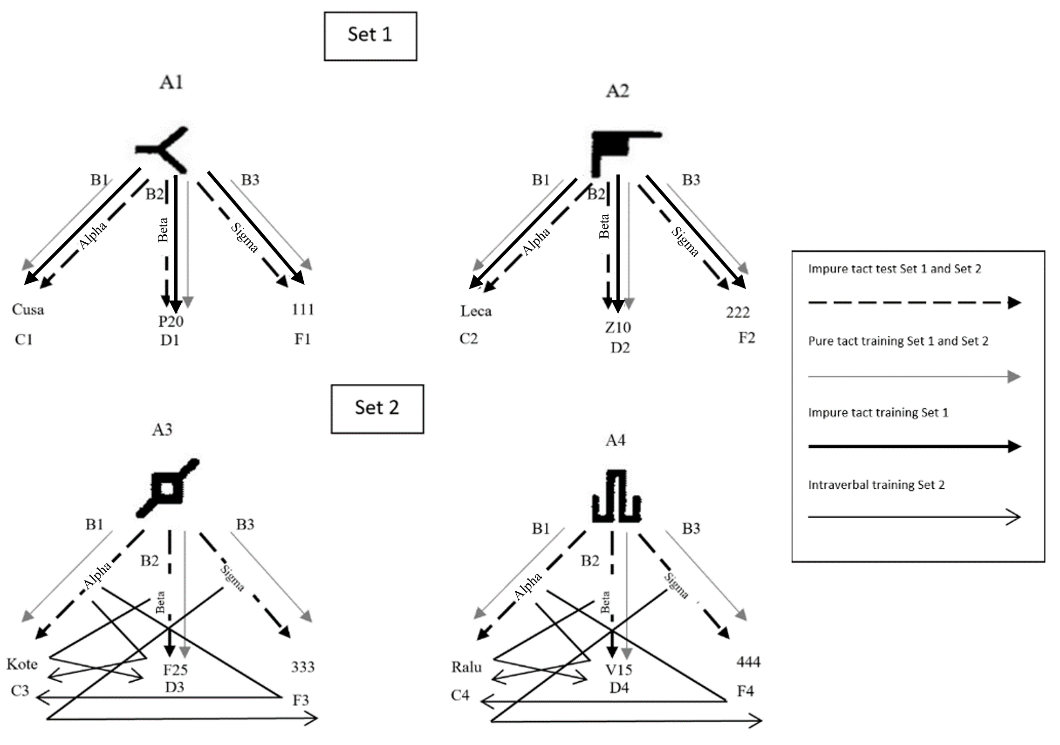


**Figure 3.** Trained relationships between pure tact and impure tact from Set 1 and pure tact and intraverbal from Set 2 (solid lines) and tested relationships (dashed lines) during Group 3.

**Results**

A repeated measures Mixed Anova was performed in which Mauchly's assumption of sphericity was assumed (*p* > .05). Regarding the equality of variances or Levene's test for the intra-subjects factors (Set) the significance level is *p*>.05, specifically for the pre-test of Set 1 (*p*= .753), pre-tests of Set 2 (*p*=.327), post-test of Set 1 (*p*=.117) and for the post-test of Set 2 (*p*= .595) so equality of variances is assumed.

These data can be seen in Table 4 below.

**Table 4.** Multiple comparisons of groups with respect to impure tact emergence testing, intragroup analysis

| Group | Set (tests) | | *p* |
| --- | --- | --- | --- |
| Group 1 | Pre-test Set 1 | Pre-test Set 2 | 1.000 |
|  |  | Post-test Set 1 | .007** |
|  |  | Post-test Set 2 | .000*** |
|  | Pre-test Set 2 | Post-test Set 1 | .042* |
|  |  | Post-test Set 2 | .000*** |
|  | Post-test Set 1 | Post-test Set 2 | .006** |
| Group 2 | Pre-test Set 1 | Pre-test Set 2 | 1.000 |
|  |  | Post-test Set 1 | 1.000 |
|  |  | Post-test Set 2 | 1.000 |
|  | Pre-test Set 2 | Post-test Set 1 | .564 |
|  |  | Post-test Set 2 | .261 |
|  | Post-test Set 1 | Post-test Set 2 | 1.000 |
| Group 3 | Pre-test Set 1 | Pre-test Set 2 | 1.000 |
|  |  | Post-test Set 1 | .000*** |
|  |  | Post-test Set 2 | .000*** |
|  | Pre-test Set 2 | Post-test Set 1 | .000*** |
|  |  | Post-test Set 2 | .000*** |
|  | Post-test Set 1 | Post-test Set 2 | .729 |

*p* < .05*; *p* < .01**; *p* < .001***

The individual results for each participant in the experiment can be found in tables A1, A2 and A3 below.

**Appendix A**

**Table A1.** Participants' results for each phase of Group 1

| Group 1 | | | | | | | | | | | | | | | |
| --- | --- | --- | --- | --- | --- | --- | --- | --- | --- | --- | --- | --- | --- | --- | --- |
|  |  | Phase | | Consequences | Trials | Participants | | | | | | | | | |
|  | | |  | | | P1 | P2 | P3 | P4 | P5 | P6 | P7 | P8 | P9 | P10 |
| Pre- tests Set 1 and Set 2 | | | | | | | | | | | | | | | |
| Set 1  Impure tact | | | *1. (AB)-RX* | No | 12 | 1/12 | 2/12 | 3/12 | 2/12 | 2/12 | 3/12 | 1/12 | 0/12 | 2/12 | 4/12 |
| Set 2  Impure tact | | | *2. (AB)-RX* | No | 12 | 5/12 | 0/12 | 2/12 | 2/12 | 3/12 | 1/12 | 7/12 | 3/12 | 2/12 | 1/12 |
| Training 1 - Set 1 | | | | | | | | | | | | | | | |
| Pure tact | | | 3. A-RC | Yes | 10 | 10 | 10 | 10 | 10 | 10 | 10 | 10 | 10 | 10 | 10 |
|  |  |  | 4. A-RD | Yes | 10 | 10 | 10 | 10 | 10 | 10 | 10 | 10 | 10 | 10 | 10 |
|  |  |  | 5. A-RF | Yes | 10 | 10 | 10 | 10 | 10 | 10 | 10 | 10 | 10 | 10 | 10 |
| Intraverbals | | | 6. (B2C)-RD | Yes | 10 | 10 | 10 | 10 | 10 | 10 | 10 | 10 | 10 | 10 | 10 |
|  |  |  | 7. (B1D)-RC | Yes | 10 | 10 | 10 | 10 | 10 | 10 | 10 | 10 | 10 | 10 | 10 |
|  |  |  | 8. (B3C)-RF | Yes | 10 | 10 | 10 | 10 | 10 | 10 | 10 | 10 | 10 | 10 | 10 |
|  |  |  | 9. (B1F)-RC | Yes | 10 | 10 | 10 | 10 | 10 | 10 | 10 | 10 | 10 | 10 | 10 |
| Total trials presented | | | | | | 70 | 70 | 70 | 70 | 70 | 70 | 70 | 70 | 70 | 70 |
| Post-tests Set 1 | | | | | | | | | | | | | | | |
| Impure tact | | | *10. (AB)-RX* | No | 12 | 4/12 | 3/12 | 11/12 | 2/12 | 4/12 | 12/12 | 4/12 | 10/12 | 4/12 | 4/12 |
| Training 2 - Set 2 | | | | | | | | | | | | | | | |
| Pure tact | | | 11. A-RC | Yes | 10 | 10 | 10 | 10 | 10 | 10 | 10 | 10 | 10 | 10 | 10 |
|  |  |  | 12. A-RD | Yes | 10 | 10 | 10 | 10 | 10 | 10 | 10 | 10 | 10 | 10 | 10 |
|  |  |  | 13. A-RF | Yes | 10 | 10 | 10 | 10 | 10 | 10 | 10 | 10 | 10 | 10 | 10 |
| Impure tact | | | 14. A3B-RX3 | Yes | 12 | 20 | 12 | 12 | 12 | 22 | 12 | 12 | 12 | 12 | 17 |
|  |  |  | 15. A4B-RX4 | Yes | 12 | 12 | 12 | 12 | 12 | 12 | 12 | 12 | 12 | 12 | 12 |
| Total trials presented | | | | | | 62 | 54 | 54 | 54 | 65 | 54 | 54 | 54 | 54 | 59 |
| Post-tests Set 2 | | | | | | | | | | | | | | | |
| Impure tact | | | *16. (AB)-RX* | No | 12 | 12/12 | 12/12 | 12/12 | 1/12 | 6/12 | 12/12 | 10/12 | 12/12 | 7/12 | 11/12 |

**Table A2.** Participant results for each phase of Group 2

| Group 2 | | | | | | | | | | | | | | |
| --- | --- | --- | --- | --- | --- | --- | --- | --- | --- | --- | --- | --- | --- | --- |
|  |  | Phase | Consequences | Trials | Participants | | | | | | | | | |
|  | |  | | | P11 | P12 | P13 | P14 | P15 | P16 | P17 | P18 | P19 | P20 |
| Pre- tests Set 1 and Set 2 | | | | | | | | | | | | | | |
| Set 1  Impure tact | | *1. (AB)-RX* | No | 12 | 4/12 | 2/12 | 3/12 | 3/12 | 2/12 | 2/12 | 4/12 | 1/12 | 4/12 | 3/12 |
| Set 2  Impure tact | | *2. (AB)-RX* | No | 12 | 2/12 | 1/12 | 1/12 | 3/12 | 2/12 | 3/12 | 3/12 | 1/12 | 2/12 | 0/12 |
| Training 1 - Set 1 | | | | | | | | | | | | | | |
| Pure tact | | 3. A-RC | Yes | 10 | 10 | 10 | 10 | 10 | 10 | 10 | 10 | 10 | 10 | 10 |
|  |  | 4. A-RD | Yes | 10 | 10 | 10 | 10 | 10 | 10 | 10 | 10 | 10 | 10 | 10 |
|  |  | 5. A-RF | Yes | 10 | 10 | 10 | 10 | 10 | 10 | 10 | 10 | 10 | 10 | 10 |
| Intraverbal | | 6. (B2C)-RD | Yes | 10 | 10 | 10 | 10 | 10 | 10 | 10 | 10 | 10 | 10 | 10 |
|  |  | 7. (B1D)-RC | Yes | 10 | 10 | 10 | 10 | 10 | 10 | 10 | 10 | 10 | 10 | 10 |
|  |  | 8. (B3C)-RF | Yes | 10 | 10 | 10 | 10 | 10 | 10 | 10 | 10 | 10 | 10 | 10 |
|  |  | 9. (B1F)-RC | Yes | 10 | 10 | 10 | 10 | 10 | 10 | 10 | 10 | 10 | 10 | 10 |
| Total trials presented | | | | | 70 | 70 | 70 | 70 | 70 | 70 | 70 | 70 | 70 | 70 |
| Post-tests Set 1 | | | | | | | | | | | | | | |
| Impure tact | | *10. (AB)-RX* | No | 12 | 9/12 | 5/12 | 5/12 | 1/12 | 4/12 | 2/12 | 6/12 | 4/12 | 1/12 | 0/12 |
| Training 2 - Set 2 | | | | | | | | | | | | | | |
| Pure tact | | 11. A-RC | Yes | 10 | 10 | 10 | 10 | 10 | 10 | 10 | 10 | 10 | 10 | 10 |
|  |  | 12. A-RD | Yes | 10 | 10 | 10 | 10 | 10 | 10 | 10 | 10 | 10 | 10 | 10 |
|  |  | 13. A-RF | Yes | 10 | 10 | 10 | 10 | 10 | 10 | 10 | 10 | 10 | 10 | 10 |
| Intraverbal | | 14. (B2C)-RD | Yes | 10 | 10 | 10 | 10 | 10 | 10 | 10 | 10 | 10 | 10 | 10 |
|  |  | 15. (B1D)-RC | Yes | 10 | 10 | 10 | 10 | 10 | 10 | 10 | 10 | 10 | 10 | 10 |
|  |  | 16. (B3C)-RF | Yes | 10 | 10 | 10 | 10 | 10 | 10 | 10 | 10 | 10 | 10 | 10 |
|  |  | 17. (B1F)-RC | Yes | 10 | 10 | 10 | 10 | 10 | 10 | 10 | 10 | 10 | 10 | 10 |
| Total trials presented | | | | | 70 | 70 | 70 | 70 | 70 | 70 | 70 | 70 | 70 | 70 |
| Post-tests Set 2 | | | | | | | | | | | | | | |
| Impure tact | | *18. (AB)-RX* | No | 12 | 11/12 | 4/12 | 12/12 | 1/12 | 10/12 | 4/12 | 3/12 | 1/12 | 1/12 | 0/12 |

**Table A3.** Participant results for each phase of Group 3

| Group 3 | | | | | | | | | | | | | |
| --- | --- | --- | --- | --- | --- | --- | --- | --- | --- | --- | --- | --- | --- |
|  | Phase | Consequences | Trials | Participants | | | | | | | | | |
|  |  | | | P21 | P22 | P23 | P24 | P25 | P26 | P27 | P28 | P29 | P30 |
| Pre- tests Set 1 and Set 2 | | | | | | | | | | | | | |
| Set 1  Impure tact | *1. (AB)-RX* | No | 12 | 1/12 | 3/12 | 3/12 | 2/12 | 1/12 | 2/12 | 4/12 | 3/12 | 1/12 | 4/12 |
| Set 2  Impure tact | *2. (AB)-RX* | No | 12 | 2/12 | 1/12 | 3/12 | 1/12 | 2/12 | 1/12 | 0/12 | 6/12 | 4/12 | 1/12 |
| Training 1 - Set 1 | | | | | | | | | | | | | |
| Pure tact | 3. A-RC | Yes | 10 | 10 | 10 | 10 | 10 | 10 | 10 | 10 | 10 | 10 | 10 |
|  | 4. A-RD | Yes | 10 | 10 | 10 | 10 | 10 | 10 | 10 | 10 | 10 | 10 | 10 |
|  | 5. A-RF | Yes | 10 | 10 | 10 | 10 | 10 | 10 | 10 | 10 | 10 | 10 | 10 |
| Impure tact | 6. A1B-RX1 | Yes | 12 | 19 | 12 | 12 | 16 | 12 | 16 | 12 | 12 | 20 | 12 |
|  | 7. A2B-RX2 | Yes | 12 | 12 | 12 | 12 | 12 | 12 | 12 | 12 | 12 | 12 | 12 |
| Total trials presented | | | | 61 | 54 | 54 | 58 | 54 | 58 | 54 | 54 | 62 | 54 |
| Post-tests Set 1 | | | | | | | | | | | | | |
| Impure tact | *8. (AB)-RX* | No | 12 | 12/12 | 12/12 | 12/12 | 12/12 | 12/12 | 12/12 | 12/12 | 12/12 | 12/12 | 4/12 |
| Training 2 - Set 2 | | | | | | | | | | | | | |
| Pure tact | 9. A-RC | Yes | 10 | 10 | 10 | 10 | 10 | 10 | 10 | 10 | 10 | 10 | 10 |
|  | 10. A-RD | Yes | 10 | 10 | 10 | 10 | 10 | 10 | 10 | 10 | 10 | 10 | 10 |
|  | 11. A-RF | Yes | 10 | 10 | 10 | 10 | 10 | 10 | 10 | 10 | 10 | 10 | 10 |
| Intraverbals | 12. (B2C)-RD | Yes | 10 | 10 | 10 | 10 | 10 | 10 | 10 | 10 | 10 | 10 | 10 |
|  | 13. (B1D)-RC | Yes | 10 | 10 | 10 | 10 | 10 | 10 | 10 | 10 | 10 | 10 | 10 |
|  | 14. (B3C)-RF | Yes | 10 | 10 | 10 | 10 | 10 | 10 | 10 | 10 | 10 | 10 | 10 |
|  | 15. (B1F)-RC | Yes | 10 | 10 | 10 | 10 | 10 | 10 | 10 | 10 | 10 | 10 | 10 |
| Total trials presented | | | | 70 | 70 | 70 | 70 | 70 | 70 | 70 | 70 | 70 | 70 |
| Post-tests Set 2 | | | | | | | | | | | | | |
| Impure tact | *16. (AB)-RX* | No | 12 | 4/12 | 12/12 | 12/12 | 10/12 | 12/12 | 12/12 | 11/12 | 10/12 | 12/12 | 1/12 |
